# Epinephrine, inodilator, or no inotrope in venoarterial extracorporeal membrane oxygenation implantation: a single-center experience

**DOI:** 10.1186/s13054-019-2605-4

**Published:** 2019-09-18

**Authors:** Viviane Zotzmann, Jonathan Rilinger, Corinna N. Lang, Klaus Kaier, Christoph Benk, Daniel Duerschmied, Paul M. Biever, Christoph Bode, Tobias Wengenmayer, Dawid L. Staudacher

**Affiliations:** 1 Faculty of Medicine, Department of Cardiology and Angiology I, Heart Center Freiburg University, University of Freiburg, Hugstetterstrasse 55, 79106 Freiburg, Germany; 2 Faculty of Medicine, Department of Medicine III (Interdisciplinary Medical Intensive Care) Medical Center, University of Freiburg, Freiburg, Germany; 3 Faculty of Medicine, Institute for Medical Biometry and Statistics, University of Freiburg, Freiburg, Germany; 4 Faculty of Medicine, Department of Cardiovascular Surgery, Heart Center Freiburg University, University of Freiburg, Freiburg, Germany

**Keywords:** Epinephrine, Inodilator, Inotropy, Venoarterial extracorporeal membrane oxygenation (VA-ECMO), Extracorporeal cardiopulmonary resuscitation (eCPR), Extracorporeal life support (ECLS), Outcome

## Abstract

**Background:**

Venoarterial extracorporeal membrane oxygenation (VA-ECMO) can be a rescue therapy for patients in cardiogenic shock or in refractory cardiac arrest. After cannulation, vasoplegia and cardiac depression are frequent. In literature, there are conflicting data on inotropic therapy in these patients.

**Methods:**

Analysis of a retrospective registry of all patients treated with VA-ECMO in a university hospital center between October 2010 and December 2018 for cardiogenic shock or extracorporeal cardiopulmonary resuscitation (eCPR) with a focus on individual early inotropic therapy.

**Results:**

A total of 231 patients (age 58.6 ± 14.3, 29.9% female, 58% eCPR, in-house survival 43.7%) were analyzed. Of these, 41.6% received no inotrope therapy within the first 24 h (survival 47.9%), 29.0% received an inodilator (survival 52.2%), and 29.0% received epinephrine (survival 25.0%). Survival of patients with epinephrine was significantly worse compared to other patient groups when evaluating 30-day survival (*p* = 0.034/*p* = 0.005) and cumulative incidence of in-hospital death (*p* = 0.001). In a multivariate logistic regression analysis, treatment with epinephrine was associated with mortality in the whole cohort (OR 0.38, *p* = 0.011) as well as after propensity score matching (OR 0.24, *p* = 0.037). We found no significant differences between patients with inodilator treatment and those without.

**Conclusion:**

Early epinephrine therapy within the first 24 h after cannulation for VA-ECMO was associated with poor survival compared to patients with or without any inodilator therapy. Until randomized data are available, epinephrine should be avoided in patients on VA-ECMO.

## Introduction

In the case of cardiogenic shock or in the context of cardiopulmonary resuscitation, venoartrial extracorporeal membrane oxygenation (VA-ECMO) is employed for hemodynamic stabilization [1]. Although randomized trials evaluating VA-ECMO in cardiogenic shock are still lacking [2], observational studies indicate beneficial effects on prognosis in patients with cardiogenic shock in acute heart failure with decreased myocardial function [3] and in refractory cardiac arrest [4]. Since a diseased myocardial function is common after cardiac arrest or in cardiogenic shock, a substantial amount of patients with VA-ECMO have decreased myocardial function [5].

VA-ECMO therapy by design leads to an increase in the afterload [6, 7], which might have a negative effect on left ventricular (LV) performance [8] and can increase LV and atrial filling pressures, pulmonary edema, LV distension, or even stasis in the pulmonary circulation [9, 10]. Because of lethal complications in case of clotting, stasis in pulmonary circulation and the left cardiac chambers has to be avoided [11, 12]. Inotropic therapy can increase LV performance and thereby overcome VA-ECMO-induced stasis [13].

Myocardial function after resuscitation and or myocardial infarction however is frequently depressed—a phenomenon which is coined as stunned or hibernating myocardium [14] and may be reversible within the first days. If inotropes should be given to these patients (and if so, which agent) is discussed controversially in the literature. Arguments against inotropes are based on pathophysiology, with a disturbance of cardiomyocyte calcium homeostasis which is critically involved in myocardial stunning. Clinically, the stunning is characterized by a decreased responsiveness of the contractile proteins to calcium and an excitation-contraction uncoupling defect [14]. The calcium-sensitizing inotrope levosimendan [15], which has been approved for the treatment of acutely decompensated heart failure, might therefore be a potential therapeutic option to improve myocardial function in stunned myocardium [15]. Regarding the use of levosimendan in patients with VA-ECMO, there are data suggesting levosimendan have beneficial outcome effects and positive effects on VA-ECMO weaning [16]. Distelmaier et al. [17] showed improved short-term and long-term survival in a retrospective registry of VA-ECMO parents with levosimendan treatment. In addition, patients treated with levosimendan were more successfully weaned from ECMO despite a more pronounced risk profile, which was reflected in a higher SAPS-3 and EuroSCORE. However, data on the effects of levosimendan therapy on survival are not consistent [18].

Next to levosimendan, dobutamine is another therapeutic option for inotropic support. The SURVIVE trial did however show equal mortality rates when dobutamine was compared to levosimendan [19].

The third inotropic agent could be epinephrine. However, there are plenty of data showing that, although cardiac output and mean arterial pressure can be reliably increased, there is a higher incidence of refractory cardiogenic shock [20]. This is consistent with the data from a large cohort of patients with cardiogenic shock, which suggested that epinephrine is associated with a threefold increase in the risk of death [21].

In conclusion, there is uncertainty which inotropic agent may be most beneficial in patients treated with VA-ECMO during the myocardial stunning phase. Therefore, we performed a retrospective analysis comparing the outcome of patients regarding the early use of levosimendan, dobutamine, and epinephrine.

## Methods

### Study setting

The study includes all adult patients after VA-ECMO implantation due to cardiogenic shock or extracorporeal cardiopulmonary resuscitation (eCPR). All data were collected retrospectively from a tertiary referral university hospital between October 2010 and December 2018. Patients after non-traumatic out-of-hospital cardiac arrest (OHCA) as well as patients after non-postoperative intra-hospital cardiac arrest (IHCA) were included. Patients who died within the first 24 h after cannulation were excluded in this research. For OHCA, eCPR cannulation was performed in-hospital after transport with ongoing manual CPR or using a mechanical chest compression device (LUCAS2, Physio Control, Neuss, Germany). The decision to cannulate was driven by team decision including at least one ECMO specialist (intensivist or cardiologist) at the bedside. Implantation was performed either in the catheterization laboratory, in the emergency room, or in the intensive care unit. By local standard, patients after OHCA with shockable primary rhythm, ST-segment elevation myocardial infarction (STEMI), or other clinical signs like chest pain before collapse (indicating a cardiac cause for collapse) are routed directly in the catheterization laboratory. Patients without non-shockable rhythm were routed to the emergency room. After VA-ECMO implantation, further diagnostic steps, including a CT scan in most patients, were directed by the responsible intensivists following current guidelines written for patients with return of spontaneous circulation (ROSC) without ECMO.

### ECMO management

The VA-ECMO therapy was initiated in patients with prolonged ongoing cardiopulmonary resuscitation without return of spontaneous circulation or in patients with severe therapy-resistant shock as indicated by the ELSO guidelines for adult cardiac failure. The indication for ECLS was stratified by the underlying disease. Cannulation for VA-ECMO was performed predominantly bi-femoral in Seldinger’s technique without primary surgical cut down by two experienced intensivists and one perfusionist. Typical venous (draining) cannulas were 21–23 Fr (French = Charrière) in diameter while arterial (returning) cannulas were 15–17 Fr. All components of the extracorporeal oxygenation system were coated with heparin. For patients without life-threatening bleeding, anticoagulation was provided by intravenous unfractionated heparin aiming at a partial thromboplastin time of 50–60 s. Mechanical ventilation was reduced during ECMO support. Peak airway pressures were aimed below 25 cmH_2_O; respiratory tidal volumes were adjusted between 4 and 6 ml/kg optimal body weight aiming at an oxygen partial pressures of 60–80 mmHg and a fractional carbon partial pressures of 35–45 mmHg as described earlier [22].

The management of vasopressors (and fluid therapy) was driven by clinical judgment of the ECMO-experienced intensivist in charge. An indication for administration of inotropic agents was to secure LV ejection thereby decreasing the risk of intra-cardial stasis. While treatment of VA-ECMO patients is strongly guided by standard operation procedures at our institution, no recommendation on positive inotropic therapy could be made. The decision to administer dobutamine, epinephrine, levosimendan, or a combination of these was driven by clinical judgment and decision-making of the experienced intensivist in charge. All patients in the levosimendan group received levosimendan 12.5 mg in 500 ml glucose 5%, given as a continuous infusion over less than 24 h without an initial bolus according to the local standard protocol.

### Data analysis and group allocation

Data presented derives from a single-center retrospective registry analysis and was blinded to patient identity and covered by an ethics approval (Ethics Committee of Albert-Ludwigs University of Freiburg, file numbers 525/17 and 151/14). For data analysis, SPSS (version 23, IBM Statistics), Prism (version 5, GraphPad), and Stata (version 15.1, StataCorp) were employed. For statistical analysis, unpaired *t* test, Fisher’s exact test, Gray test, and Wald test were used as applicable, and a *p* value of ≤ 0.05 was considered statistically significant. Data are given as [mean ± standard deviation] or [odds ratio (OR), 95% confidence interval (CI)] if not stated otherwise.

Groups were formed according to inotropic therapy given within the first 24 h after cannulation for VA-ECMO. All patients with continuous epinephrine infusion (with or without dobutamine or levosimendan) were grouped in the “epinephrine group” (group C). All patients without epinephrine but with either dobutamine, levosimendan, or a combination of both were grouped in the “dobutamine/levosimendan or inodilator group” (group B). Patients without epinephrine, dobutamine, or levosimendan within the first 24 h were grouped in the “no inotropy group” (group A). Patients were excluded when survival was below 24 h and when patients could not be reliably stratified into one of the three groups. As for 30-day survival, all patients dismissed from our hospital alive before reaching 30 days of hospitalization were considered 30-day survivors. Mode of death has been categorized according to Witten et al. [23]. Propensity score matching was performed using SPSS with a nearest neighbor matching algorithm and a caliper of 0.1. Matching was performed for predictors of hospital survival available during the first 24 h (as detected by the multivariate logistic regression analysis of hospital survival in the whole cohort (age, eCPR as indication for VA-ECMO, and gender, see Fig. 4) as well as for known predictors of survival (a shockable first rhythm and lactate levels 24 h after cannulation). All factors were known at the time of treatment and might have influenced the physician’s decision to treat a patient with different inotropic agents. Cumulative incidence curves were calculated using competing risk regression according to the Fine and Gray method [24] with discharge alive from the hospital as a competing event.

## Results

### Study population

**Fig. 1:**
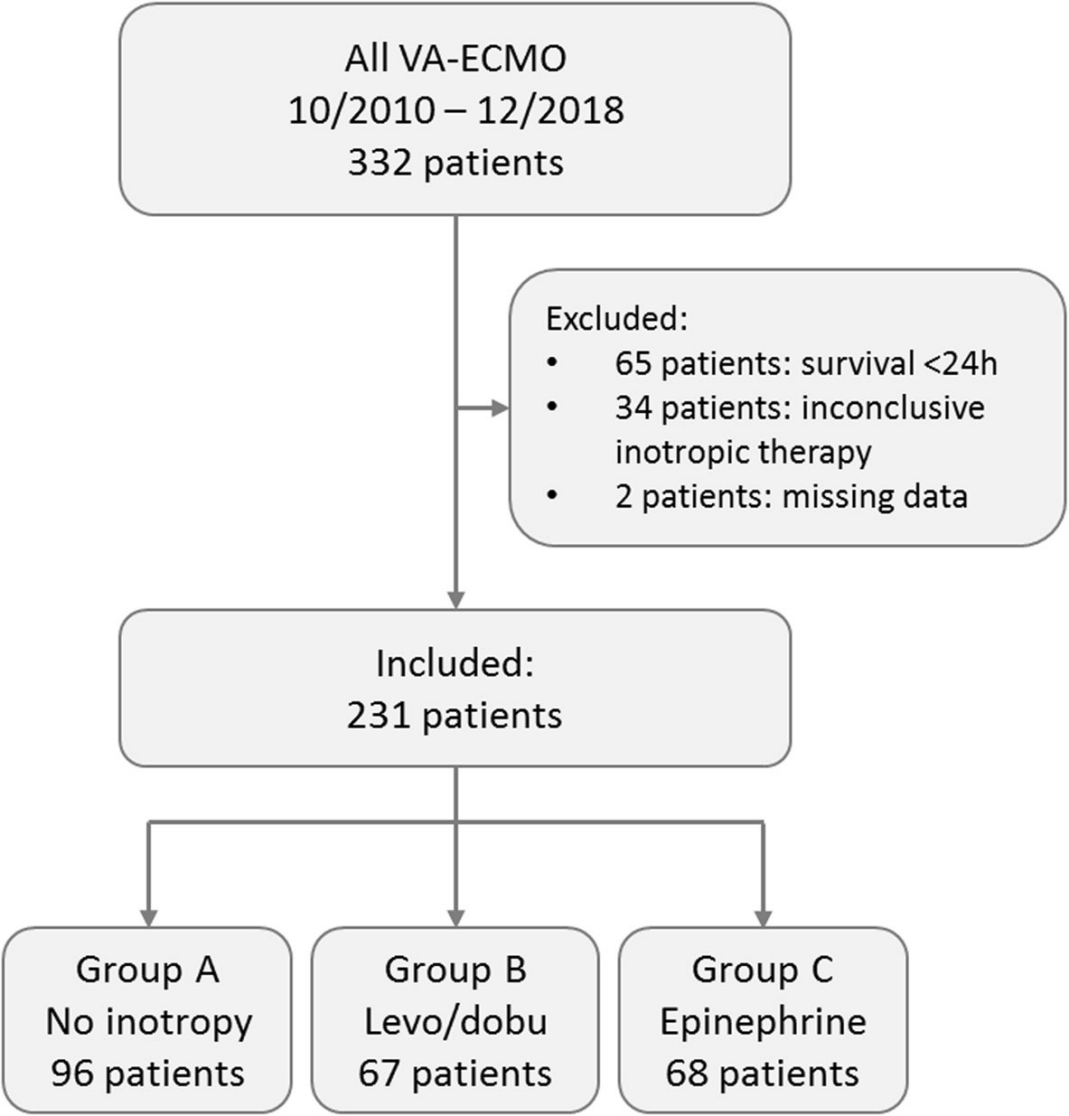
Flow chart of study population. A total of 231 patients could be analyzed for the present study. VA-ECMO, venoarterial extracorporeal membrane oxygenation; dob, dobutamine; lev, levosimendan

Between October 2010 and December 2018, 332 patients were treated with a VA-ECMO due to a cardiogenic shock or resuscitation without return of spontaneous circulation. After exclusion of 101 patients, a total of 231 patients were evaluated in this research (for reasons of exclusion, see Fig. 1). Patients included were at a median age of 58.58 ± 14.27 years, and 29.9% were female. Reasons for VA-ECMO implantations were extracorporeal cardiopulmonary resuscitation (eCPR) in 58% or cardiogenic shock (mostly due to STEMI or NSTEMI in 25.9% and 25.5%, respectively). Patient characteristics are given in Table 1 and in Additional file 1.

**Table 1:** Patients’ characteristics and outcome

	Overall		A: no inotropic		B: inodilator pooled		C: epinephrine pooled		*p* value		
	Absolute	%	Absolute	%	Absolute	%	Absolute	%	A vs B vs C	A vs C	B vs C
Number of patients	231	100	96	41.6	67	29.0	68	29.4			
No. of flow time [min]	1.74	± 3.55	1.75	± 3.26	0.96	± 2.56	2.56	± 4.48	0.106	0.353	0.041
Mean age [years]	58.58	± 14.27	58.32	± 15.77	59.82	± 13.81	57.72	± 12.5	0.680	0.795	0.358
Female gender	69	29.9	29	30.2	23	34.3	17	25.0	0.494	0.486	0.262
In-house survival	96	41.6	46	47.9	35	52.2	17	25.0	*0.002*	*0.003*	*0.001*
Alive after > 30 days	87	37.7	43	44.8	35	52.2	19	28.4	*0.013*	*0.034*	*0.005*
Neurological withdrawal	47	20.3	20	20.8	7	10.4	17	25.0	0.083	0.572	0.041
Co-morbidities	28	12.1	7	7.3	7	10.4	9	13.2	0.451	0.286	0.791
Cardiogenic shock/instability	61	26.4	21	21.9	16	23.9	22	32.3	0.296	0.152	0.339
Respiratory failure	7	3.0	4	4.2	2	29.8	1	1.5	0.611	0.404	0.619
Presumed patient will	1	0.4	1	1.0	0	0	0	0	0.400	1.0	1.0
ECMO-data											
VA-ECMO rotation (rounds/min)	2685	± 663	2662	± 542	2760	± 552	2677	± 659	0.550	0.876	0.456
VA-ECMO blood flow (l/min)	3.75	± 1.08	3.53	± 1.08	3.78	± 1.08	3.85	± 1.08	0.240	0.091	0.553
Continuous norepinephrine infusion	209	90.5	83	86.5	60	89.6	66	97.1	0.071	0.026	0.096
ECPR	133	57.6	50	54.2	34	50.7	46	68.7	0.078	*0.054*	*0.055*
Scores											
SOFA score	14.47	± 2.61	14.80	± 2.62	14.22	± 2.89	14.59	± 2.29	0.177	0.647	0.418
SAPS2 score	48.69	± 15.00	49.09	± 15.70	46.58	± 14.78	50.19	± 14.18	0.657	0.647	0.150
SAVE score	− 6.23	± 5.28	− 5.96	± 5.16	− 5.27	± 5.5	− 7.54	± 4.98	0.670	*0.051*	*0.013*
Primary rhythm											
PEA/asystolia/non-shockable	144	62	68	70.8	35	52.2	41	61.2	*0.050*	0.181	0.388
VT/VF/shockable	70	30	22	22.9	24	35.8	24	34.3	0.120	0.112	1.000
Unknown primary rhythm	17	7	6	6.3	8	13.6	3	4.5	0.212	0.737	0.128
Reason of ECLS implantation											
Cardiogenic shock	183	79.2	72	75.0	60	89.6	56	83.6	0.062	0.339	0.323
Other shock	48	20.8	24	25.0	7	10.5	12	16.4	0.062	0.339	0.323
Risk factors											
Coronary heart disease	172	74.4	66	68.7	56	83.6	50	73.5	0.100	0.602	0.209
Hypertension	95	41.1	40	41.6	36	53.7	19	27.9	*0.010*	0.098	*0.003*
Peripheral artery disease	17	7.3	6	6.3	7	10.4	4	5.9	0.515	1.000	0.365
Lung disease	30	13.0	15	15.6	7	10.4	8	11.8	0.588	0.649	1.000
Diabetes mellitus	55	23.9	23	23.6	20	29.9	12	17.6	0.250	0.439	0.109

Characteristics of patients included in the registry are given as the number of patients (percent of group) or as mean ± standard deviation. Significance is calculated between all the groups or between the epinephrine and either the inodilator or no inotropy group

### Inotropic and vasopressor therapy

**Fig. 2:**
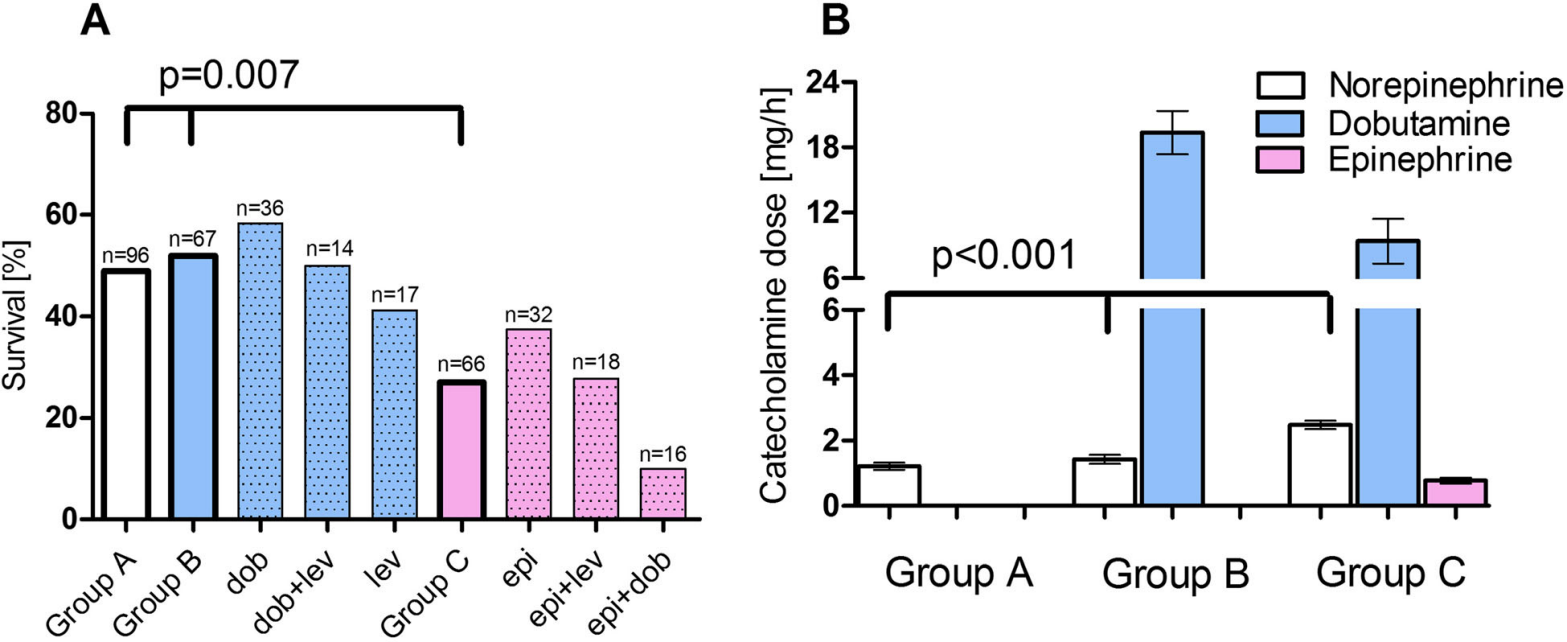
Catecholamine therapy in venoarterial extracorporeal membrane oxygenation 24 h after cannulation. **a** Survival after venoarterial extracorporeal membrane oxygenation implantation in patients without any positive inotropic therapy (group A), in patients with dobutamine/levosimendan (composite shown as group B, blue dotted columns give different combinations), and in patients with epinephrine (group C as pink column, different combination given in dotted columns). There was a significant difference in the outcome in patients on different inotropic agent with epinephrine performing worse than groups A and B. dob, dobutamine; lev, levosimendan; epi, epinephrine. **b** Mean dose of catecholamines 24 h after cannulation of VA-ECMO. As shown in the white bars, patients in group C had significantly higher norepinephrine doses compared to group A or group B. By design, there was a significant difference in dobutamine (blue columns) and epinephrine (pink columns) doses given

**Fig. 3:**
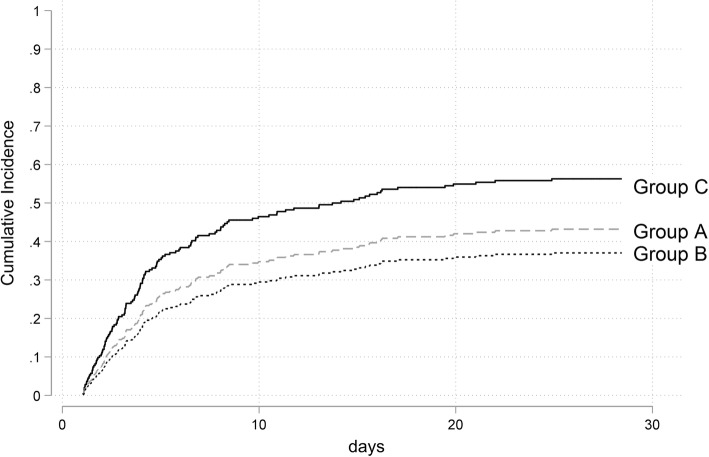
Cumulative incidence of in-hospital death. Cumulative incidence curves of hospital mortality after cannulation for venoarterial extracorporeal membrane oxygenation with hospital discharge as a competing event. Patients with epinephrine (black line, group C) perform significantly worse than patients with either no inotropic therapy (gray line, group A, subdistribution hazard ratio 0.52, *p* = 0.001) or dobutamine/levosimendan (dotted line, group B, subdistribution hazard ratio 0.44, *p* < 0.001)

Average VA-ECMO blood flow was similar between the groups (*p* = 0.240, Table 1). The 30-day survival was evaluated in different subgroups stratified by early inotropic therapy used. When considering no early inotropic therapy as baseline, there was no significant difference between patients with dobutamine, levosimendan, or the combination of both and patients without early inotropic therapy. The worst outcome was detected in patients treated with epinephrine with or without a combination of dobutamine or levosimendan (Fig. 2a). This difference in survival was confirmed by the cumulative incidence of hospital death curve as given in Fig. 3. When evaluating the concomitant vasopressor therapy, there was a significant difference in norepinephrine dose between the groups with the highest vasopressor doses being detected in the epinephrine group (Fig. 2b).

### Predictors of survival

**Fig. 4:**
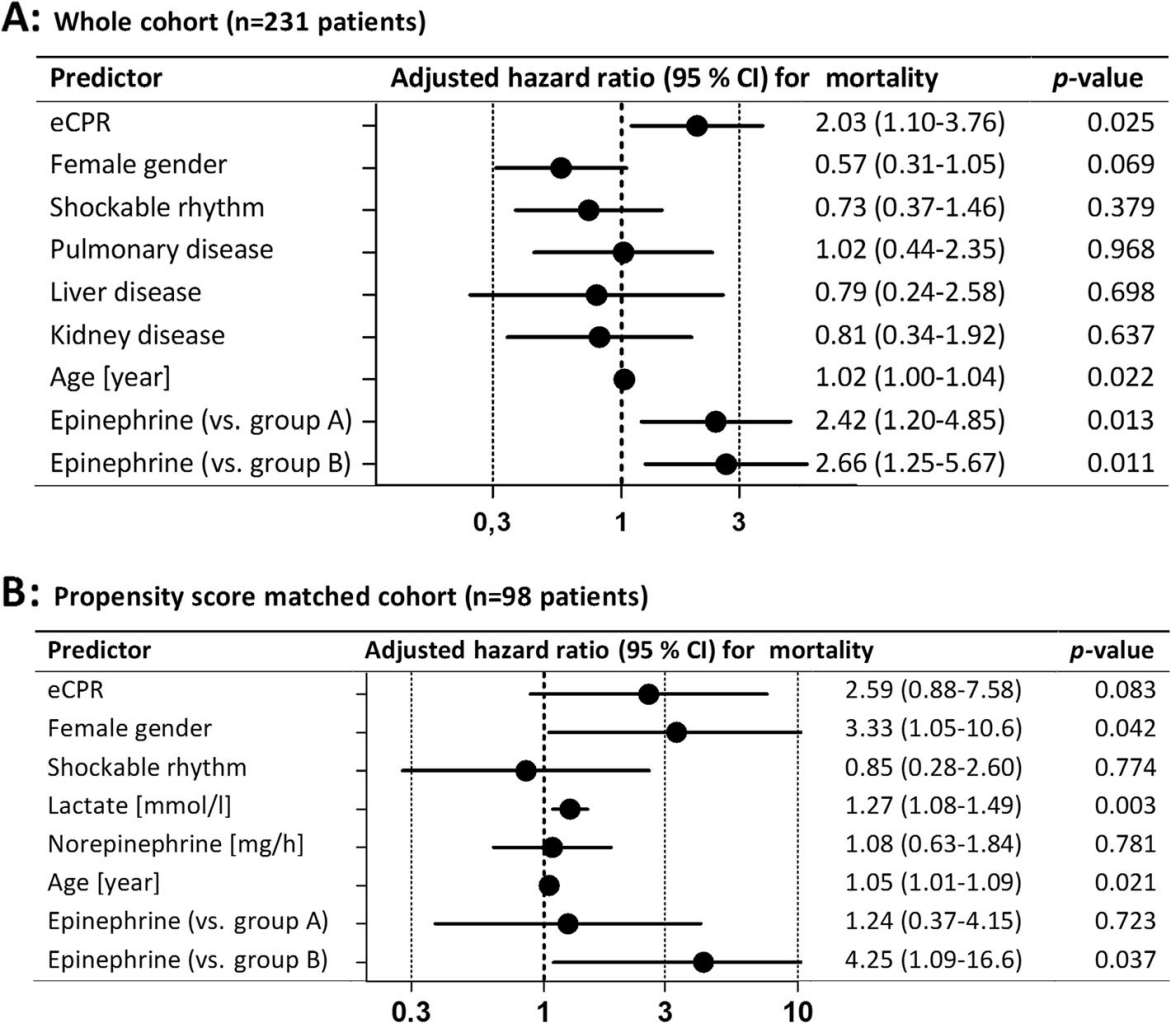
Multivariate logistic regression analyses of predictors of 30-day mortality. Forest plot giving predictors of 30 mortality of the whole cohort (top) and in the propensity score match cohort (bottom). Epinephrine treatment was an independent predictor of worse outcome compared to patients without any positive inotropic therapy (group A) as well as compared to patients with dobutamine/levosimendan (group B) in the whole group. After propensity score matching, epinephrine was still an independent predictor of worse outcome compared to group B

Factors that were associated with survival to 30 days were tested in a multivariate logistic regression analysis. In the whole cohort, implantation during eCPR, age, and epinephrine use were significant and independent predictors of poor outcome as given in Fig. 4a. After propensity score matching for items given in the “Methods” section, we were able to match 49 patients in the epinephrine group (group C) with 49 patients without epinephrine. In a multivariate logistic regression analysis of the matched cohort, age and epinephrine were strong independent predictors of survival alongside female gender and lactate, as demonstrated in Fig. 4b.

## Discussion

In this retrospective registry study, patients after cannulation for VA-ECMO on continuous epinephrine infusion within the first day performed significantly worse compared to patients either on dobutamine or levosimendan therapy or compared to patients without any positive inotropic therapy.

This reduced prognosis was confirmed by analyzing the cumulative incidence of death in a hospital with hospital discharge as a competing event, after adjustment for confounders in a multivariate logistic regression analysis as well as in a propensity score-matched cohort.

To our best knowledge, only animal studies evaluate epinephrine therapy in the context of VA-ECMO. In a randomized pig trial of eCPR in ischemic refractory ventricular fibrillation, pigs randomized to epinephrine had a worse prognosis not reaching statistical significance [25]. While data on epinephrine in patients on ECMO is limited, there are data from patients without extracorporeal support showing no benefit or even a signal of harm of epinephrine in patients during cardiopulmonary resuscitation [26, 27] or cardiogenic shock [23]. This might be explained by the various adverse effects of epinephrine treatment including an increase in lactate levels by pyruvate generation through a cAMP-dependent mechanism [28, 29] and an increase in cardiac double products [20] in cardiogenic shock patients. This adverse correlation of epinephrine treatment and hospital mortality in non-VA-ECMO patients is confirmed by large registries of acute heart failure after propensity score matching [30].

Therefore, it has been suggested to use norepinephrine as the first-line inopressor in cardiogenic shock [31]. There are data, however, that in patients with severe cardiogenic shock (defined as need of a vasopressor) without ECMO, the combination of an inodilator and an inopressor is associated with a significant increase in short-term survival when compared to an inopressor alone [32]. In our VA-ECMO collective, there was no difference in short-term survival when comparing positive inotropic therapy (with dobutamine, levosimendan, or a combination of both) with no inotropic therapy within the first 24 h. This observation is in concordance with the data from randomized trials in patients after cardiac surgery and persistent need for mechanical support which showed no significant improvement by implementation of a levosimendan treatment to the catecholamine mix used [33]. There are data on device-supported ECMO weaning including intra-aortic balloon pump or the Impella pump [34]. It is unclear if our findings can be extrapolated to these patients. There are data form registries however suggesting an improved ECMO weaning and survival in levosimendan-treated patients on ECMO [16, 17]. Given the limited patient numbers in our registry, we cannot comment on the outcome in the inodilator subgroups.

## Limitations

Several limitations of the present study have to be considered when interpreting the results presented in this manuscript. First of all, the observational and retrospective design makes the data prone to bias since all treatment decisions were made by the intensivist in charge without randomization. The number of patients undergoing eCPR was numerically higher in the epinephrine group compared to the other groups not reaching statistical significance, which suggests a potential bias with sicker patients in the epinephrine group. Also, norepinephrine co-therapy is a confounder of the results presented here since patients on epinephrine received more norepinephrine compared to patients in the other groups. Even if the studied groups were comparable by the other patient characteristics and the disease severity scores and homogenized by propensity score matching, findings presented here have to be considered hypothesis generating only.

## Conclusion

Patients after cannulation for VA-ECMO on continuous epinephrine infusion within the first day performed significantly worse compared to patients with or without inodilator therapy. Until randomized data are available, epinephrine should be avoided in patients on VA-ECMO.

## Supplementary information

**Additional file 1: Table S1.** Mode of death. **Table S2.** Baseline characteristics of the matched cohort. **Table S3.** In-House Survival in groups only including patients with continuous epinephrine infusion. **Table S4.** Characteristics of the different groups.

## Data Availability

The datasets used for this study are available from the corresponding author on request.

## Abbreviations

eCPR: Extracorporeal cardiopulmonary resuscitation
CPR: Cardiopulmonary resuscitation
ROSC: Return of spontaneous circulation
VA-ECMO: Venoartrial extracorporeal membrane oxygenation
OHCA: Out-of-hospital cardiac arrest
IHCA: Intra-hospital cardiac arrest
STEMI: ST-segment elevation myocardial infarction
NSTEMI: Non-ST-segment elevation myocardial infarction
