# Supplementary material for: Epinephrine, inodilator, or no inotrope in venoarterial extracorporeal membrane oxygenation implantation: a single-center experience
Source: Crit Care. 2019 Sep 18;23:320. doi: 10.1186/s13054-019-2605-4 (PMC6751670; doi:10.1186/s13054-019-2605-4)
Supplement: Supplementary file 1 — Additional file 1: Table S1. Mode of death. Table S2. Baseline characteristics of the matched cohort. Table S3. In-House Survival in groups only including patients with continuous epinephrine infusion. Table S4. Characteristics of the different groups. [file 13054_2019_2605_MOESM1_ESM.docx]

**Electronic supplemental material**

**Epinephrine, inodilator or no inotrope in venoarterial extracorporeal membrane oxygenation implantation - a single center experience**

Viviane Zotzmann^1,2^, Jonathan Rilinger^1,2^, Corinna N. Lang^1,2^, Klaus Kaier^4^, Christoph Benk^3^**,** Daniel Duerschmied^1,2^, Paul M. Biever^1,2^, Christoph Bode^1,2^, Tobias Wengenmayer^1,2^, Dawid L. Staudacher^1,2^

Table S1: mode of death

| **Mode of death in all ECLS- patients** | | | | | | | | | | | |
| --- | --- | --- | --- | --- | --- | --- | --- | --- | --- | --- | --- |
|  | **Overall** | | **A:**  **No inotropic** | | **B:**  **Inodilator pooled** | | **C:**  **Epinephrine pooled** | | **D:**  **Levosimendan mono** | | **E:**  **Dobutamine mono** |
| **Number of patients** | 231 (100%) | 96 (42%) | | 67 (29%) | | 68 (29%) | | 17 (7.4%) | | 35 (15%) | |
| **alive after > 30 day** | 87 (37.7%) | 43 (44.8%) | | 35 (52%) | | 19 (28.4%) | | 6 (35.3%) | | 20 (57.1%) | |
| **neurological withdrawal** | 47 (20.3%) | 20 (20.8%) | | 7 (10%) | | 17 (25.0%) | | 2 (11.8%) | | 4 (11.4%) | |
| **co-morbidities** | 28 (12.1%) | 7 (7.3%) | | 7 (10%) | | 9 (13.2%) | | 2 (11.8%) | | 5 (14.3%) | |
| **cardiogenic shock /instability** | 61 (26.4%) | 21 (21.9%) | | 16 (24%) | | 22 (32.3%) | | 6 (35.3%) | | 5 (14.3%) | |
| **respiratory failure** | 7 (3.0%) | 4 (4.2%) | | 2 (30%) | | 1 (1.5%) | | 1 (5.9%) | | 1 (2.9%) | |
| **presumed patient will** | 1 (0.4%) | 1 (1.0%) | | 0 (0%) | | 0 (0.0%) | | 0 (0.0%) | | 0 (0.0%) | |

| **Mode of death ECPR- patients** | | | | | | | | | | | | |
| --- | --- | --- | --- | --- | --- | --- | --- | --- | --- | --- | --- | --- |
|  | **Overall** | | **A:**  **No inotropic** | | **B:**  **Inodilator pooled** | | **C:**  **Epinephrine pooled** | | **D:**  **Levosimendan mono** | | **E:**  **Dobutamine mono** | |
| **Number of patients** | | 132 (100%) | | 50 (37.8%) | | 35 (26.5%) | | 47 (30%) | | 9 (6.8%) | | 18 (13.6%) |
| **In-house-survival** | | 44 (33.3%) | | 19 (38.0%) | | 16 (45.7%) | | 9 (19.1%) | | 3 (33.3%) | | 8 (44.4%) |
| **SAVE Score** | | -6.23 | | -5.96 | | -8 | | -7.54 | | -6.88 | | -4.72 |
| **SAPS2 Score** | | 48.69 | | 49.09 | | 48 | | 50.19 | | 49.00 | | 45.76 |
| **SOFA Score** | | 14.47 | | 14.8 | | 13.7 | | 14.59 | | 13.41 | | 14.50 |
| **neurological withdrawal** | | 33 (25.0%) | | 12 (24.0%) | | 6 (17.1%) | | 15 (31.9%) | | 1 (11.1%) | | 4 (22.2%) |
| **cardiogenic shock /instability** | | 45 (34.1%) | | 15 (30.0%) | | 12 (34.3%) | | 18 (38.3%) | | 4 (44.4%) | | 4 (22.2%) |
| **other** | | 10 (7.6%) | | 4 (8.0%) | | 4 (11.4%) | | 5 (10.6%) | | 0 (0.0%) | | 1 (5.6%) |

Table S2: baseline characteristics of the matched cohort

|  | Overall | Epinephrine | No Epinephrine | No inotropy | Inodilator | P value  2 vs 3 |
| --- | --- | --- | --- | --- | --- | --- |
| No. of patients | 98 | 49 | 49 | 29 | 20 |  |
| 30 day survival | 40 (40.8 %) | 17 (34.7 %) | 23 (46.9 %) | 11 (37.9 %) | 12 (60 %) | 0.3041 |
| eCPR | 66 (67.3 %) | 32 (65.3 %) | 34 (69.4 %) | 21 (72.4 %) | 13 (65 %) | 0.8297 |
| Female gender | 31 (31.6 %) | 14 (28.6 %) | 17 (34.7 %) | 8 (27.6 %) | 9 (45 %) | 0.6644 |
| Lactate mmol/l | 6 ± 4.6 | 6.1 ± 4.4 | 5.9 ± 4.9 | 6.2 ± 4.1 | 5.3 ± 5.9 | 0.8339 |
| Shockable initial rhythm | 31 (31.6 %) | 15 (30.6 %) | 16 (32.7 %) | 7 (24.1 %) | 9 (45 %) | 1 |
| Age [years] | 58.1 ± 13.9 | 58.8 ± 13.3 | 57.3 ± 14.6 | 56.2 ± 14.5 | 59 ± 14.9 | 0.6131 |
| Norepinephrine | 2.1 ± 1.2 | 2.4 ± 1.1 | 1.7 ± 1.2 | 1.5 ± 1.2 | 2 ± 1.1 | 0.0017 |

Table S3: In-House Survival in groups only including patients with continuous epinephrine infusion

|  | no inotropy  all patients | Inodilator  all patients | Epinephrine  all patients | p-value | p-value | p-value |
| --- | --- | --- | --- | --- | --- | --- |
| Patients | 96 | 67 | 68 | 1 vs 2 vs 3 | 1 vs 3 | 2 vs 3 |
| Survival [%] | 47.9% | 52.2% | 26.5% |  |  |  |
| Survivors | 46 | 35 | 18 | 0.0044 | 0.0060 | 0.0027 |

|  | no inotropy with cont. norepinephrine | Inodilator  with cont. norepinephrine | Epinephrine with cont. norepinephrine | p-value | p-value | p-value |
| --- | --- | --- | --- | --- | --- | --- |
| Patients | 83 | 60 | 66 | 1 vs 2 vs 3 | 1 vs 3 | 2 vs 3 |
| Survival [%] | 48.2% | 51.7% | 27.3% |  |  |  |
| Survivors | 40 | 31 | 18 | 0.0090 | 0.0113 | 0.0062 |

Table 4: Characteristics of the different groups

| **Characteristics** | **Overall** | | **A: No inotropic** | | **B: Inodilator pooled** | | **C: Epinephrine pooled** | | **D: Levosimendan monotherapy** | | **E: Dobutamine monotherapy** | |
| --- | --- | --- | --- | --- | --- | --- | --- | --- | --- | --- | --- | --- |
|  | absolute | % | absolute | **%** | absolute | **%** | absolute | % | absolute | % | absolute | % |
| Number of patients | 231 | 100 | 96 | 41.6 | 67.0 | 29.0 | 68 | 29.4 | 17 | 7.4 | 35 | 15.2 |
| No-flow time [min] | 1.74 | ±3.6 | 1.75 | ±3.3 | 0.96 | ±2.6 | 2.56 | ±4.5 | 0.94 | ±2.5 | 0.96 | ±2.7 |
| Mean age [years] | 58.58 | ±14.3 | 58.32 | ±15.8 | 59.82 | ±13.8 | 57.72 | ±12.5 | 60.72 | ±13.9 | 59.51 | ±13.9 |
| Female gender | 69 | 29.9 | 29 | 30.2 | 23.0 | 34.3 | 17 | 25.0 | 5 | 29.4 | 13 | 37.1 |
| In-house- survival | 96 | 41.6 | 46 | 47.9 | 35.0 | 52.2 | 17 | 25.0 | 7 | 41.2 | 20 | 57.1 |
| **ECMO-data:** |  |  |  |  |  |  |  |  |  |  |  |  |
| VA-ECMO Rotation  (rounds per minute) | 2685.0 | ±663 | 2662.0 | ±542 | 2760 | ±552 | 2677.0 | ±659 | 2948.00 | ±591 | 2678.56 | ±588 |
| VA-ECMO Blood-flow  (liter per minute) | 3.8 | ±1.08 | 3.5 | ±1.08 | 3.78 | ±1.08 | 3.9 | ±1.08 | 3.99 | ±1.04 | 3.71 | ±1.19 |
| Continuous norepine-  phrine infusion | 209 | 90.5 | 83 | 86.5 | 60.0 | 89.6 | 66 | 97.1 | 16 | 94.1 | 32 | 91.4 |
| ECPR | 133 | 57.6 | 50 | 54.2 | 34.0 | 50.7 | 46 | 68.7 | 8 | 47.1 | 18 | 51.4 |
| **Scores:** |  |  |  |  |  |  |  |  |  |  |  |  |
| SOFA – score | 14.47 | ±2.6 | 14.8 | ±2.6 | 14.22 | ±2.9 | 14.59 | ±2.3 | 14.57 | ±2.6 | 14.50 | ±2.7 |
| SAPS2 – score | 48.69 | ±15.0 | 49.09 | ±15.7 | 46.58 | ±14.8 | 50.19 | ±14.3 | 48.69 | ±15.0 | 45.76 | ±14.5 |
| SAVE – Score | -6.23 | ±5.3 | -5.96 | ±5.2 | -5.27 | 5.5 | -7.54 | ±5.0 | -6.23 | ±5.2 | -4.72 | ±5.5 |
| **Primary ryhthm:** |  |  |  |  |  |  |  |  |  |  |  |  |
| PEA /Asystolia / not-  shockable | 144 | 62.3 | 68 | 70.8 | 35.0 | 52.2 | 41 | 61.2 | 9 | 52.9 | 21 | 60.0 |
| VT / VF /shockable | 70 | 30.3 | 22 | 22.9 | 24.0 | 35.8 | 24 | 34.3 | 8 | 47.1 | 9 | 25.7 |
| Unknown primary rhythm | 17 | 7.4 | 6 | 6.3 | 8.0 | 13.6 | 3 | 4.5 | 0 | 0.0 | 5 | 14.3 |
| **Risk factors:** |  |  |  |  |  |  |  |  |  |  |  |  |
| Coronary heart disease | 172.0 | 74.4 | 66.0 | 68.7 | 56 | 83.6 | 50 | 73.5 | 15 | 88.2 | 26 | 74.3 |
| Hypertension | 95.0 | 41.1 | 40.0 | 41.6 | 36 | 53.7 | 19 | 27.9 | 9 | 52.9 | 19 | 54.3 |
| Peripheral artery disease | 17.0 | 7.3 | 6.0 | 6.3 | 7 | 10.4 | 4 | 5.9 | 3 | 17.6 | 3 | 8.6 |
| Lung disease | 30.0 | 13.0 | 15.0 | 15.6 | 7 | 10.4 | 8 | 11.8 | 0 | 0.0 | 5 | 14.3 |
| Diabetes mellitus | 55.0 | 23.9 | 23.0 | 23.6 | 20 | 29.9 | 12 | 17.6 | 5 | 29.4 | 12 | 34.3 |
| **Reason of ECLS-Implantation:** |  |  |  |  |  |  |  |  |  |  |  |  |
| Dilated Cardiomyopathy | 11 | 4.8 | 4 | 4.2 | 4.0 | 6.0 | 3 | 4.5 | 1 | 5.9 | 1 | 2.9 |
| Congenital heart failure | 3 | 1.3 | 2 | 2.1 |  |  | 1 | 1.5 |  |  | 2 | 5.7 |
| Electrical storm | 5 | 2.2 | 2 | 2.1 | 1.0 | 1.5 | 2 | 3.0 | 1 | 5.9 |  |  |
| Cardiogenic shock | 18 | 7.8 | 9 | 9.4 | 7.0 | 10.4 | 6 | 9.0 | 3 | 17.6 |  |  |
| Infarction. STEMI | 60 | 26.0 | 21 | 21.9 | 20.0 | 29.9 | 18 | 26.9 | 7 | 41.2 | 10 | 28.6 |
| Infarction. NSTEMI | 59 | 25.5 | 21 | 21.9 | 21.0 | 31.3 | 21 | 31.3 | 4 | 23.5 | 8 | 22.9 |
| Myocarditis | 4 | 1.7 | 2 | 2.1 | 2.0 | 3.0 |  |  | 1 | 5.9 | 1 | 2.9 |
| Septic shock | 10 | 4.3 | 5 | 5.2 |  |  | 5 | 7.5 |  |  |  |  |
| Valvular shock | 8 | 3.5 | 6 | 6.3 | 1.0 | 1.5 |  |  |  |  | 2 | 5.7 |
| Heart transplant graft  rejection | 1 | 0.4 | 1 | 1.0 |  |  |  |  |  |  |  |  |
| unclear / other | 13 | 5.6 | 3 | 3.1 | 3.0 | 4.5 | 2 | 3.0 |  |  | 3 | 8.6 |
| Hypo-/ Hyperthermia  accidentially | 2 | 0.9 | 1 | 1.0 | 1.0 | 1.5 | 1 | 1.5 |  |  |  |  |
| Hypo-/ Hyperkalemia | 2 | 0.9 | 1 | 1.0 |  |  |  |  |  |  | 1 | 2.9 |
| Lung failure / ARDS | 15 | 6.5 | 10 | 10.4 | 2.0 | 3.0 | 3 | 4.5 |  |  | 2 | 5.7 |
| pulmonary embolism | 14 | 6.1 | 4 | 4.2 | 4.0 | 6.0 | 5 | 7.5 |  |  | 3 | 8.6 |
| Anaphylactic shock | 1 | 0.4 |  |  |  |  | 1 | 1.5 |  |  |  |  |
| Hemorraghe shock  /hypovolemia | 3 | 1.3 | 3 | 3.1 |  |  |  |  |  |  | 1 | 2.9 |
| Intoxication | 2 | 0.9 | 1 | 1.0 | 1.0 | 1.5 |  |  |  |  | 1 | 2.9 |
